# 3-Substituted Coumarins Inhibit NorA and MepA Efflux Pumps of *Staphylococcus aureus*

**DOI:** 10.3390/antibiotics12121739

**Published:** 2023-12-15

**Authors:** José B. de Araújo-Neto, Cícera D. de M. Oliveira-Tintino, Gildênia A. de Araújo, Daniel S. Alves, Fernanda R. Ribeiro, Guilherme A. Brancaglion, Diogo T. Carvalho, Clara Mariana Gonçalves Lima, Hani S. H. Mohammed Ali, Irfan A. Rather, Mohmmad Y. Wani, Talha B. Emran, Henrique D. M. Coutinho, Valdir de Q. Balbino, Saulo R. Tintino

**Affiliations:** 1 Postgraduate Program in Biological Sciences, Biosciences Center, Federal University of Pernambuco, Recife 50740-570, PE, Brazil; jose.bezerra456@gmail.com (J.B.d.A.-N.); valdir.balbino@ufpe.br (V.d.Q.B.); 2 Laboratory of Microbiology and Molecular Biology, Department of Biological Chemistry, Regional University of Cariri, Crato 63105-000, CE, Brazil; datianemorais@hotmail.com (C.D.d.M.O.-T.); gildenia.araujo@urca.br (G.A.d.A.); daniel.sampaio10@urca.br (D.S.A.); saulorelison@gmail.com (S.R.T.); 3 Pharmaceutical Chemistry Research Laboratory, Faculty of Pharmaceutical Sciences, Federal University of Alfenas, Alfenas 37130-001, MG, Brazil; fernandareis.r@hotmail.com (F.R.R.); guiabrancaglion@gmail.com (G.A.B.); diogo.carvalho@unifal-mg.edu.br (D.T.C.); 4 Department of Food Science, Federal University of Lavras, Lavras 37203-202, MG, Brazil; 5 Department of Biological Sciences, Faculty of Science, King Abdulaziz University, Jeddah 21589, Saudi Arabia; hmohammedali@kau.edu.sa (H.S.H.M.A.); ammm@kau.edu.sa (I.A.R.); 6 Department of Chemistry, College of Science, University of Jeddah, Jeddah 21589, Saudi Arabia; mwani@uj.edu.sa; 7 Department of Pathology and Laboratory Medicine, Warren Alpert Medical School, Brown University, Providence, RI 02912, USA; talha_bin_emran@brown.edu; 8 Legorreta Cancer Center, Brown University, Providence, RI 02912, USA; 9 Department of Pharmacy, Faculty of Allied Health Sciences, Daffodil International University, Dhaka 1207, Bangladesh

**Keywords:** antibiotic resistance, fluoroquinolones, synergism, benzoyl, structure–activity relationship

## Abstract

Coumarins are compounds with scientifically proven antibacterial properties, and modifications to the chemical structure are known to improve their effects. This information is even more relevant with the unbridled advances of antibiotic resistance, where *Staphylococcus aureus* and its efflux pumps play a prominent role. The study’s objective was to evaluate the potential of synthetic coumarins with different substitutions in the C-3 position as possible inhibitors of the NorA and MepA efflux pumps of *S. aureus*. For this evaluation, the following steps took place: (i) the determination of the minimum inhibitory concentration (MIC); (ii) the association of coumarins with fluoroquinolones and ethidium bromide (EtBr); (iii) the assessment of the effect on EtBr fluorescence emission; (iv) molecular docking; and (v) an analysis of the effect on membrane permeability. Coumarins reduced the MICs of fluoroquinolones and EtBr between 50% and 87.5%. Coumarin C1 increased EtBr fluorescence emission between 20 and 40% by reinforcing the evidence of efflux inhibition. The molecular docking results demonstrated that coumarins have an affinity with efflux pumps and establish mainly hydrogen bonds and hydrophobic interactions. Furthermore, C1 did not change the permeability of the membrane. Therefore, we conclude that these 3-substituted coumarins act as inhibitors of the NorA and MepA efflux pumps of *S. aureus*.

## 1. Introduction

*Staphylococcus aureus* is a Gram-positive bacterium described as one of the most worrying pathogens for human health, contributing to increased morbidity and mortality rates [1]. This microorganism can cause anything from mild skin infections to invasive infections, such as endocarditis, bacteremia, pneumonia, and osteomyelitis [2]. In addition to pathogenicity, *S. aureus* is also relevant for the multidrug resistance acquired by its strains, which has made it a high-priority species for the development of new drugs, according to the World Health Organization [3].

Efflux pumps are proteins responsible for expelling compounds toxic to bacteria, and in strains of *S. aureus*, they are among the main mechanisms of resistance to fluoroquinolone antibiotics [4]. Relevant in this context, the efflux pumps NorA and MepA are chromosome-encoded transporters belonging to the major facilitator superfamily (MFS) and multidrug and toxic compound extrusion family (MATE), respectively [5,6]. These efflux pumps, in addition to having fluoroquinolones (e.g., norfloxacin and ciprofloxacin) as substrates, also extrude quaternary ammonium compounds (e.g., benzalkonium chloride), ethidium bromide (EtBr), pentamidine, acriflavine, and cetrimide, for example [7,8].

Given the impact of antibiotic resistance on public health (around 1.3 million deaths/year), the development of efflux pump inhibitors (EPIs) is one of the strategies used to combat bacteria resistant to multiple drugs (MDRs) [9,10]. The ability of EPIs to restore the effectiveness of existing antibiotics against MDR strains is even more prominent given the current context of the pharmaceutical industry, where investment in antibiotic research and development is decreasing despite the advancement of bacterial resistance [11,12,13].

Coumarins are compounds synthesized by plants, fungi, and bacteria and obtained via chemical synthesis [14]. Among its bioactivities, antimicrobial, anti-inflammatory, anticonvulsant, anticancer, and antioxidant activities can be mentioned [15,16]. Studies such as that by De Araújo et al. [17] and Šimunović et al. [18] provide evidence that coumarins and their derivatives can act as EPIs, including against *S. aureus* efflux pumps, such as the Nora protein.

Analysis of the structure–activity relationship of coumarins indicates that certain modifications made to the chemical structure can improve their pharmacological effects. Against bacteria, the best substitution patterns include radicals linked to carbons 3, 4, 6, 7, and 8; however, studies associate the C-3 position with the most significant results [19,20,21].

**Figure 1:**
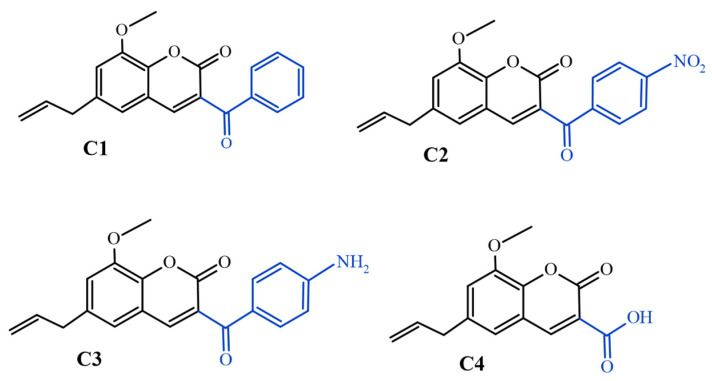
Chemical structures of coumarins. C1: 3-benzoyl-8-methoxy-6-(prop-2-en-1-yl)-2*H*-chromen-2-one; C2: 8-methoxy-3-(4-nitrobenzoyl)-6-(prop-2-en-1-yl)-2*H*-chromen-2-one; C3: 3-(4-aminobenzoyl)-8-methoxy-6-(prop-2-en-1-yl)-2*H*-chromen-2-one; C4: 8-methoxy-2-oxo-6-(prop-2-en-1-yl)-2*H*-chromene-3-carboxylic acid. Substitutions at the C-3 position are highlighted in blue.

Therefore, this study aims to evaluate the potential of synthetic coumarins with different substitutions at the C-3 position (Figure 1) as possible inhibitors of the NorA and MepA efflux pumps of *S. aureus*. Given the results obtained, we conclude that these coumarins act as EPIs in strains of *S. aureus* and coumarin with a benzoyl radical is the most promising derivative.

## 2. Results

### 2.1. Antibacterial Activity

The evaluation of intrinsic antibacterial activity showed that the coumarins tested did not have significant effects against strains 1199B and K2068 of *S. aureus*, as well as chlorpromazine (CPZ). However, carbonyl cyanide 3-chlorophenylhydrazone (CCCP) demonstrated a direct effect against the strains (Table 1).

### 2.2. Association with Antibiotics and Ethidium Bromide

**Figure 2:**
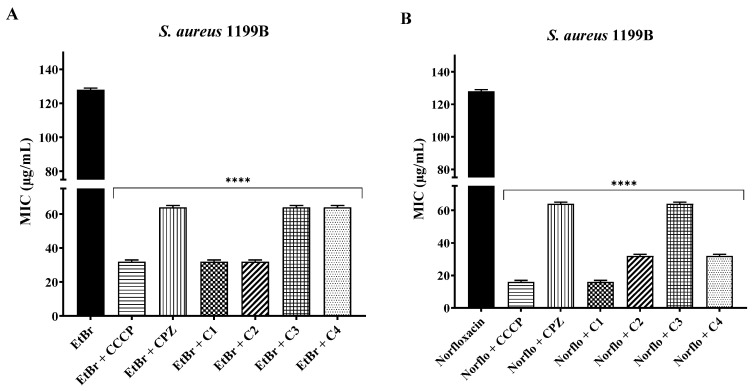
Potentiation of the effect of ethidium bromide (**A**) and norfloxacin (**B**) by 3-substituted coumarins against *S. aureus* 1199B. ****: statistical significance—*p* < 0.0001.

Against *S. aureus* 1199B, the association of coumarins in subinhibitory concentrations with EtBr resulted in synergism, with reductions of 50% (C3 and C4) and 75% (C1 and C2) in the compound’s MIC. In association with norfloxacin, coumarins also enhanced the effect of the antibiotic, reducing its MIC by 50% (C3), 75% (C2 and C4), and 87.5% (C1). In both associations, the effect of coumarins was comparable to the positive control, including CCCP, which was the most effective standard inhibitor (Figure 2).

**Figure 3:**
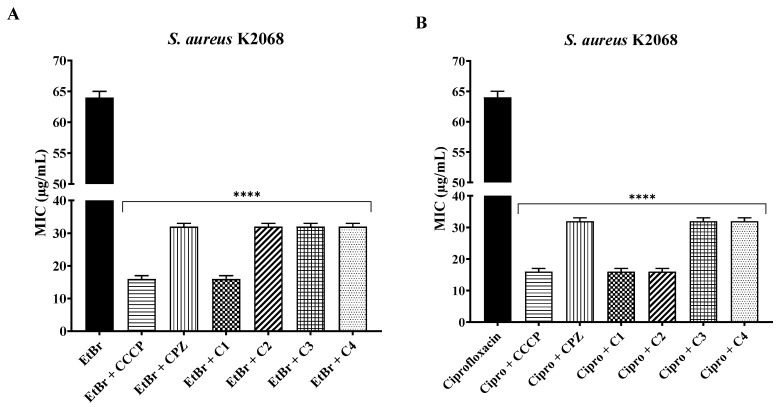
Potentiation of the effect of ethidium bromide (**A**) and ciprofloxacin (**B**) by 3-substituted coumarins against *S. aureus* K2068. ****: statistical significance—*p* < 0.0001.

Against *S. aureus* K2068, coumarins reduced the MIC of EtBr by 50% (C2, C3, and C4) and 75% (C1). Coumarins also showed MIC reductions of 50% (C3 and C4) and 75% (C1 and C2) associated with ciprofloxacin. Again, the reductions were equivalent to those observed in the positive control with CCCP and CPZ (Figure 3).

These results indicate the possibility that the enhancement of antibiotic activity by coumarins occurred through efflux pump inhibition. Furthermore, we noticed that the most effective coumarin in all associations was C1, so it was selected for the other in vitro tests.

### 2.3. Fluorescence Emission

**Figure 4:**
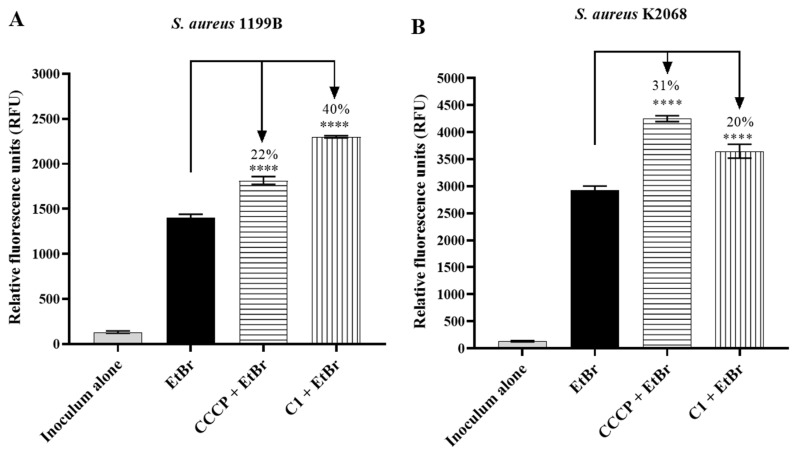
Increased ethidium bromide fluorescence emission by C1 coumarin in *S. aureus* strains 1199B (**A**) and K2068 (**B**). ****: statistical significance—*p* < 0.0001.

Fluorescence emission assays reinforced the evidence of efflux inhibition by the tested coumarins. In strain 1199B, we identified that C1 at a concentration of 10 µg/mL increased the fluorescence emission of EtBr by 40%, being more effective than CCCP, which was tested at a concentration five times higher (Figure 4A). C1 increased EtBr fluorescence by 20% at the same concentration in strain K2068; however, it was less effective than CCCP at 50 µg/mL (Figure 4B).

### 2.4. Molecular Docking

Molecular docking of coumarins with the NorA efflux pump homology model resulted in interaction energies that ranged from −99.1264 to −138.965 and MolDock scores from −89.4395 to −125.394, such that the affinity with the efflux pump followed the following order: C2 > C1 > C3 > C4. However, when calculating ligand efficiency, C4 (neutral) presented the best result, followed by C1 (Table 2).

**Figure 5:**
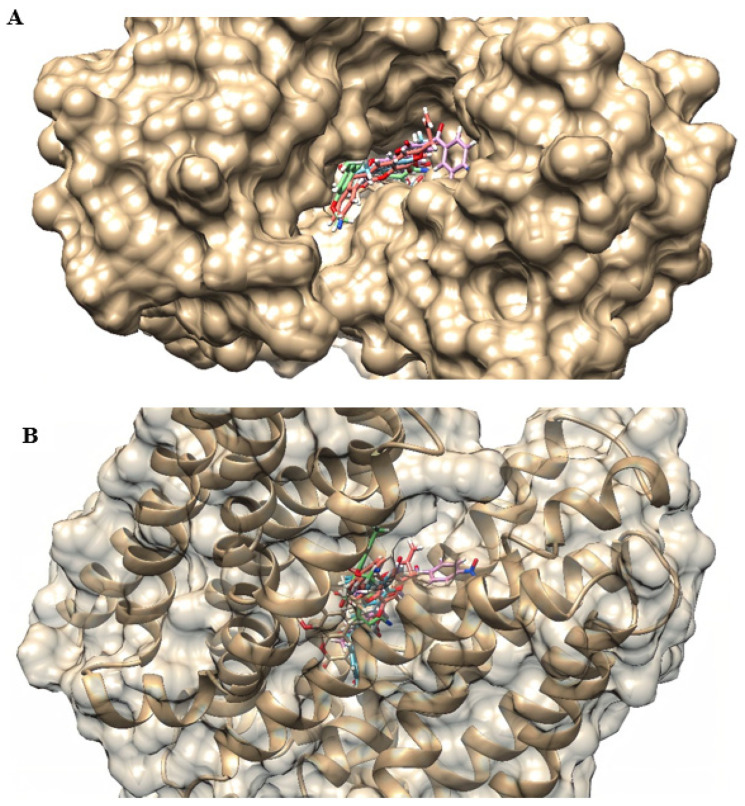
3D representation of the location of the best poses of coumarins in the NorA efflux pump binding site: (**A**) superior view; (**B**) side view. C1—blue; C2—pink; C3 (neutral)—green; C3 (ionized)—red; C4 (neutral)—beige; C4 (ionized)—white.

Figure 5 shows the location, in 3D, of the best pose of each coumarin in the efflux pump model. From this, it is clear that the different substitutions in the C-3 position influence the binding to the target, which results in differences in docking calculations. The common structures occupied similar positions, while C-3 substitutions followed different orientations. For example, we have C1 and C2, the compounds with the best interaction energies: while the benzoyl radical of C1 (in blue) is on the left, the 4-nitrobenzoyl of C2 (in pink) is facing the right.

**Figure 6:**
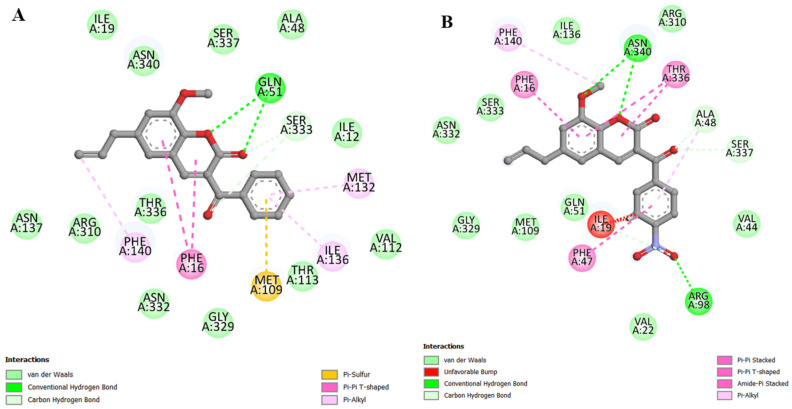
Maps of interactions, in 2D, between coumarins and the NorA efflux pump: (**A**) coumarin C1; (**B**) coumarin C2.

In the in vitro results shown in Figure 3, coumarin C1 showed the best effects, followed by C2, but in the docking results, the order was the opposite. Analyzing the interactions of C1, we have the following information: (i) van der Waals interactions (around 5 Å); (ii) hydrogen bonds with Gln51 (2.23 and 2.49 Å, respectively); (iii) carbon–hydrogen bonds with Ser333 (3.19 Å with the coumarin nucleus and 2.79 Å with the benzoyl radical); (iv) π-sulfur with Met109 (4.33 Å); (v) π-π T-shaped with Phe16 (5.31 and 5.25 Å, respectively); and (vi) π-alkyl with Phe140 (4.74 Å), Ile136 (5.30 Å), and Met132 (5.37 Å) (Figure 6A). All of these interactions are considered favorable.

Coumarin C2 has favorable interactions similar to those described for the previous derivative, with the following results being obtained: (i) van der Waals interactions (around 5 Å); (ii) hydrogen bonds with Asn340 (2.35 Å with the methoxy group and 2.32 Å with the coumarin nucleus) and Arg98 (2.20 Å); (iii) carbon–hydrogen bonds with Ala48 (3.19 Å), Ser337 (2.47 Å) and Ile19 (2.60 Å); (iv) π-π stacked with Phe47 (4.32 Å); (v) π-π T-shaped with Phe16 (5.46 Å); (vi) amide-π stacked with Thr336 (5.05 and 4.69 Å, respectively); (vii) π-alkyl with Phe140 (5.09 Å) and Ala48 (5.36 Å). However, we identified two unfavorable interactions classified as steric bumps with Ile19 (2.22 Å with the hydrogen atom and 3.10 Å with the carbon atom) (Figure 6B). These unfavorable interactions represent the primary evidence that justifies the fact that C1 had better effects in vitro than C2.

**Figure 7:**
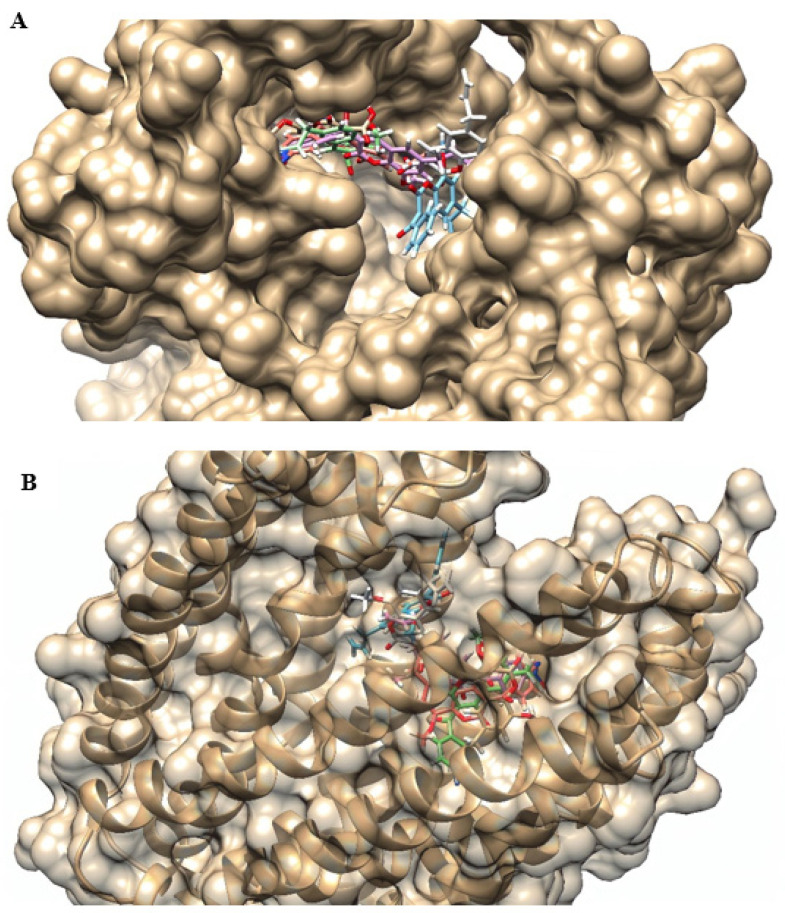
3D representation of the location of the best poses of coumarins in the MepA efflux pump binding site: (**A**) superior view; (**B**) side view. C1—blue; C2—pink; C3 (neutral)—green; C3 (ionized)—red; C4 (neutral)—beige; C4 (ionized)—white.

We also verified the affinity of coumarins with the MepA model, with interaction energies from −92.5696 to −121.81 and MolDock scores ranging from −83.2516 to −111.936. C1 and C4 demonstrated the highest and lowest affinity, respectively, while C2 and C3 showed similar values. Regarding ligand efficiency, C4 (ionized) and C1 were the derivatives with the best results (Table 3). As described in the NorA efflux pump, despite being close, the coumarin derivatives occupied different locations in the MepA efflux pump binding site because of their structural differences (Figure 7).

**Figure 8:**
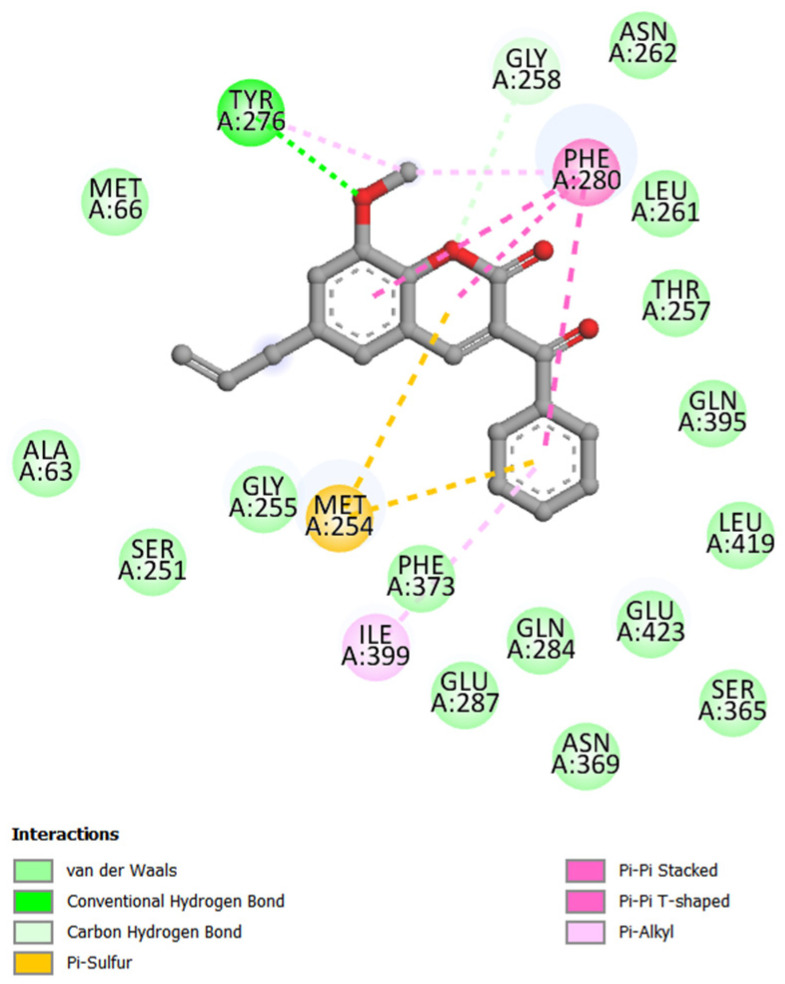
Map of interactions in 2D between coumarin C1 and the MepA efflux pump.

Coumarin C1, which had the best in silico and in vitro results, showed similar interactions to those exposed to the NorA efflux pump. Existing interactions are classified as (i) van der Waals interactions (about 5 Å); (ii) hydrogen bond with Tyr276 (2.25 Å); (iii) carbon–hydrogen bond with Gly258 (2.62 Å); (iv) π-sulfur with Met254 (5.42 Å with the coumarin nucleus and 4.91 Å with the benzoyl); (v) π-π stacked with Phe280 (with the coumarin nucleus, 4.33 and 4.01, respectively); (vi) π-π T-shaped with Phe280 (5.87 Å, with the benzoyl radical); and (vii) π-alkyl with Tyr276 (4.70 Å), Phe280 (3.94 Å), and Ile399 (5.42 Å) (Figure 8).

The results of molecular docking with the NorA and MepA models, in addition to demonstrating the differences between the four coumarins, which have the C-3 substitutions as a differential, illustrated how the same compound interacts with different proteins. With the NorA efflux pump, the propenyl group (C6 substitution) of C1 showed interaction with the amino acid, and the methoxy (C8 substitution) did not (Figure 6A). In contrast, the opposite occurred with the MepA efflux pump, with the methoxy radical interacting with different residues of the protein (Figure 8).

**Figure 9:**
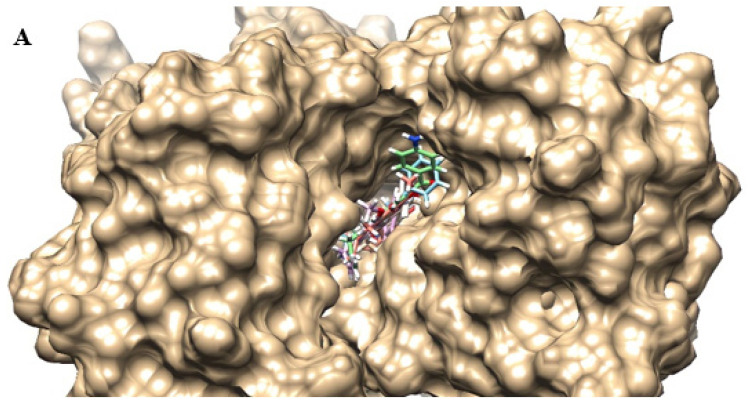

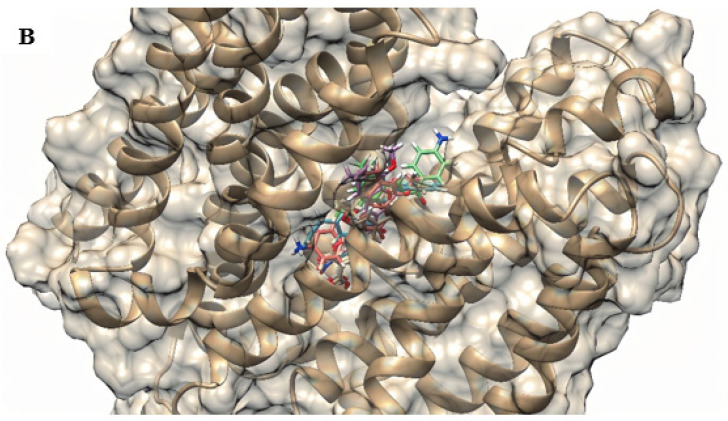
3D representation of the location of the best poses of coumarins in the NorA efflux pump binding site: (**A**) Superior view; (**B**) Side view. C1—blue; C2—pink; C3 (neutral)—green; C3 (ionized)—red; C4 (neutral)—beige; C4 (ionized)—white.

From molecular docking with ligands and receptor (NorA efflux pump), both flexible, the following binding energies were recorded: −8.6 Kcal/mol for C1, −8.7 Kcal/mol for C2, −8.5 Kcal/mol for C3 (neutral and ionized), −7.7 Kcal/mol for neutral C4, and −7.6 Kcal/mol for ionized C4. Although there is a similarity with docking only with flexible ligands in descending order of affinity, the position of coumarins in the binding site was different from the first evaluation, as demonstrated in Figure 9. For example, while C2 was in the most superior position in the first docking analysis, in this second docking, neutral C3 occupied a similar position.

**Figure 10:**
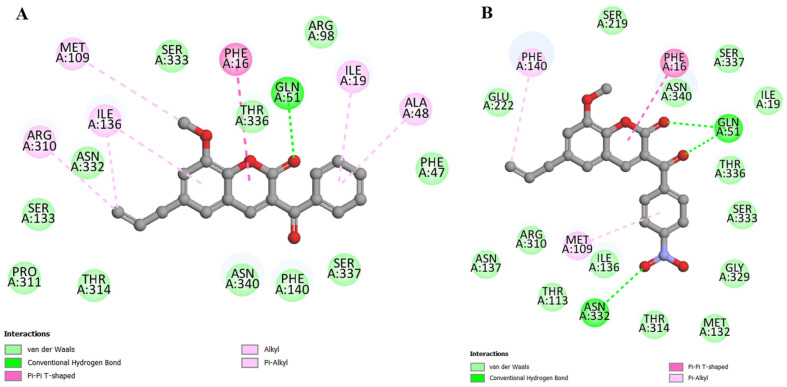
Maps of interactions, in 2D, between coumarins and the NorA efflux pump: (**A**) coumarin C1; (**B**) coumarin C2.

With coumarin C1, the following interactions occurred: (i) van der Waals interactions (around 5 Å); (ii) hydrogen bond with Gln51 (2.14 Å); (iii) π-π T-shaped with Phe16 (4.93 Å); (iv) alkyl with Arg310 (3.99 Å), Ile136 (4.96 Å with the propenyl radical), and Met109 (5.00 Å); (v) π-alkyl with Ile136 (5.37 Å with the coumarin nucleus), Ile19 (4.88 Å) and Ala48 (4.94 Å) (Figure 10A). Coumarin C2 has established the following interactions: (i) van der Waals interactions (around 5 Å); (ii) hydrogen bonds with Gln51 (2.84 Å with the coumarin nucleus and 1.85 Å with the benzoyl radical) and Asn332 (2.83 Å); (iii) π-π T-shaped with Phe16 (5.07 Å); (iv) π-alkyl with Phe140 (4.17 Å) and Met109 (5.12 Å) (Figure 10B).

**Figure 11:**
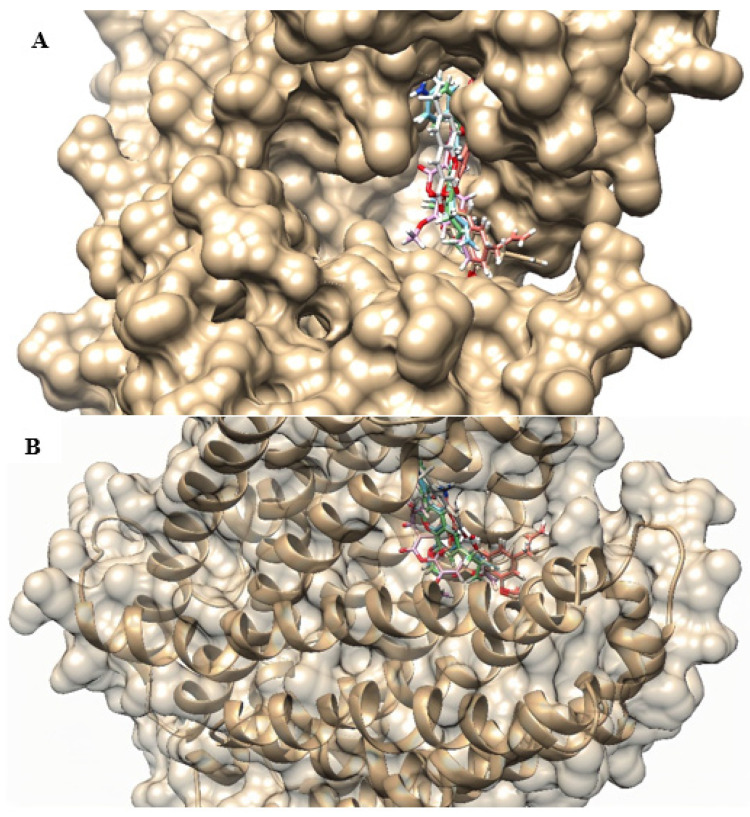
3D representation of the location of the best poses of coumarins in the MepA efflux pump binding site: (**A**) superior view; (**B**) side view. C1—blue; C2—pink; C3 (neutral)—green; C3 (ionized)—red; C4 (neutral)—beige; C4 (ionized)—white.

The binding energies recorded in the second molecular docking with the MepA model (ligands and receptor, both flexible) demonstrated a similar order of affinity to the docking with the non-flexible receptor, with the following values being recorded: −8.5 Kcal/mol for C1 and C2, −8.2 Kcal/mol for C3 (neutral and ionized), −6.7 Kcal/mol for neutral C4 and −6.5 Kcal/mol for ionized C4. In this case, coumarins C1 and C2 presented the same binding energies, unlike the previous analysis. The position of the coumarins in the binding site also showed differences, with the compounds occupying more similar positions in the efflux pump cavity (Figure 11).

**Figure 12:**
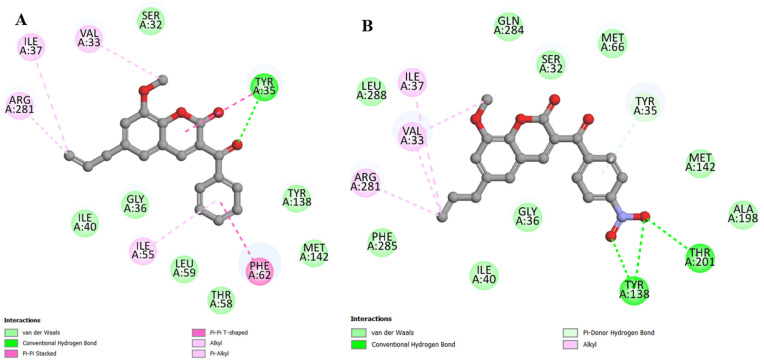
Maps of interactions in 2D between coumarins and the MepA efflux pump: (**A**) coumarin C1; (**B**) coumarin C2.

Coumarin C1 performed the following interactions with the MepA model: (i) van der Waals interactions (around 5 Å); (ii) hydrogen bond with Tyr35 (2.88 Å); (iii) π-π stacked with Phe62 (3.97 Å); (iv) π-π T-shaped with Tyr35 (5.13 Å); (v) alkyl with Arg281 (4.02 Å), Ile37 (4.79 Å) and Val33 (4.91 Å); and (vi) π-alkyl with Ile55 (5.33 Å) (Figure 12A). With coumarin C2, the following was recorded: (i) van der Waals interactions (around 5 Å); (ii) hydrogen bonds with Tyr138 (3.00 Å and 2.90 Å) and Thr201 (3.00 Å); (iii) π-donor hydrogen bond with Tyr35 (3.92 Å); and (iv) alkyl with Arg281 (4.33 Å), Ile37 (4.67 Å), and Val33 (4.30 Å with the propenyl radical and 5.06 Å with the methoxy radical) (Figure 12B).

After carrying out a second analysis via molecular docking, applying flexibility to the ligands and receptors, we noticed similarities among the compounds that demonstrated the best results in terms of energy/interaction but differences in other aspects, as demonstrated in the figures (2D and 3D): the position of the coumarins in their respective binding sites, the residues with which the coumarins established interactions, and different types of interactions with the same residue in the comparison between the first and second molecular docking. These similarities and differences apply to both the NorA and MepA models, and even with the differences, these results still corroborate what was obtained in in vitro assays.

### 2.5. Effects on Membrane Permeability

**Figure 13:**
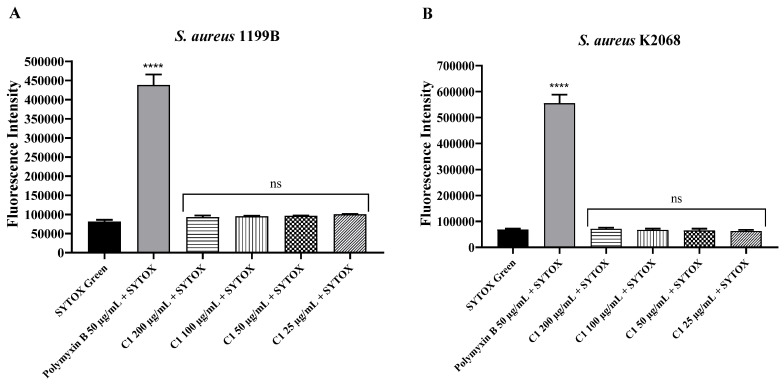
Evaluation of the effect of C1 coumarin on the fluorescence intensity of SYTOX Green in *S. aureus* 1199B (**A**) and K2068 (**B**) strains. ****: statistical significance—*p* < 0.0001; ns: not significant.

Coumarin C1, tested at concentrations of 25 to 200 μg/mL, did not increase the fluorescence emission of SYTOX Green in *S. aureus* 1199B and K2068 compared to the negative control, indicating that it did not alter membrane permeability in both strains. Conversely, polymyxin B at 50 μg/mL altered the fluorescence intensity significantly (Figure 13). These results eliminate the hypothesis that coumarins enhanced antibiotic activity by increasing drug influx.

## 3. Discussion

Coumarin derivatives with different substitutions, such as methoxy, tolyl, benzyl, and chlorine, in strategic positions (C-3, C-6, and C-7), presented MICs significantly lower than those obtained in our study, especially against Gram-positive bacteria, such as *S. aureus*, against which an MIC of just 2 μg/mL was recorded [22]. The study by Tiwari et al. [23], who evaluated the antibacterial activity of synthetic coumarins with a hydroxyl at C-4 and different C-3 substitutions, also reported the potential of these substances, which inhibited *S. aureus* at concentrations of 50 to 78 μg/mL.

There are coumarins with relevant antibacterial effects, and examples of this are the antibiotics novobiocin, chlorobiocin, and coumermycin A1, which are naturally synthesized by Streptomyces spp. and have action, for example, against resistant *S. aureus* [24]. Like fluoroquinolones, these substances prevent the synthesis of nucleic acids by inhibiting the enzyme DNA gyrase [25]. Furthermore, coumarins inhibit protein synthesis and bacterial metabolism [26].

As mentioned, specific substitution patterns increase the activity of coumarins against bacteria; however, this parameter is not absolute, as the added radicals also impact the activity, and the magnitude of the effect varies according to the target species [27]. As in our study, research by Sovari et al. [28] and Ge et al. [29] found that 3- and 7-substituted coumarins did not inhibit the growth of Gram-negative and Gram-positive bacteria, including *S. aureus*. For this research, the absence of intrinsic effects was positive, as EPIs should not have antibacterial activity to avoid selecting resistant strains [30].

Despite not having direct action against bacteria, certain coumarins enhance the effect of antibiotics against MDR strains. Coumarin derivatives evaluated by Martin et al. [31], with MICs ≥ 1024 μg/mL, increased fluoroquinolone and aminoglycoside activity against *S. aureus* and *Escherichia coli*. Coumarins with the same C-3 substitutions as the compounds evaluated in the present study showed synergism with antibiotics. Against *S. aureus*, coumarins with benzoyl and 4-nitrobenzoyl radicals caused the most significant potentiation of norfloxacin, corroborating our results [32].

Against strains of *S. aureus* carrying the NorA, TetK, and MsrA efflux pumps, coumarins isolated from Rutaceae species increased the activity of the antibiotics norfloxacin, tetracycline, and erythromycin, respectively, and imperatorin, the most effective compound, reduced the MIC of EtBr against strain 1199B (NorA) [33]. In the study by Martin et al. [34], 4-, 7- and 8-substituted coumarins reduced the MICs of ciprofloxacin and EtBr against *S. aureus* K2068, a carrier of the MepA efflux pump. The effect of coumarins was equal to or greater than the chlorpromazine control in both studies.

As described in the results (Section 2.2) and the research mentioned above, the joint potentiation of antibiotics and EtBr is considered evidence of the inhibition of efflux pumps. However, more than this, synergism is needed to state that coumarins acted as EPIs, making it necessary to use other methods. The fluorescence emission or accumulation test used in the present research is one of the primary methods for investigating EPIs. The increase in fluorescence occurs because of the more significant intracellular accumulation of EtBr caused by the inhibition of efflux [35].

Coumarins from *Mesua ferrea*, evaluated by Roy et al. [36], in addition to enhancing the activity of norfloxacin and EtBr against *S. aureus* 1199B, increased the accumulation of EtBr, but with lower efficacy than the verapamil control. Galbanic acid showed synergism in association with ciprofloxacin and EtBr in resistant isolates of *S. aureus* and increased the intensity of EtBr fluorescence more significantly than the same EPI [37]. These results reinforce the potential of coumarins as EPIs and the effects arising from structural differences and target microorganisms.

Molecular docking analyses showed that the most abundant interactions between coumarins and efflux pump models were hydrogen bonds (conventional or carbon–hydrogen bonds), hydrophobic and van der Waals interactions, and π-sulfur interactions to a lesser extent. Despite the scarcity of in silico studies between coumarins and efflux pumps, it is possible to see similarities in the interactions established by these compounds.

With the NorA efflux pump, osthol potentiated the antibiotic activity against *S. aureus* 1199B and had in silico affinity with the protein, highlighting the hydrophobic interactions and hydrogen bonds, for example, with Arg98, as occurred with coumarin C2 [38]. Furanochromones are compounds structurally similar to coumarins, and the study by Rodrigues et al. [39] showed that khellin, like C1, interacts with the NorA model through hydrophobic interactions with Phe16 and Ile136. The amino acids Arg310, Asn340, Gln51, Met109, Ser333, and Ser337 also appear in both studies but are involved in different types of interactions.

Martin et al. [34] obtained interaction energies ranging from −92.3 to −124.6 Kcal/mol in the analysis of coumarin derivatives with the MepA model. 7-(alpha-D-Galactopyranosyloxy)-4-methyl-2*H*-1-benzopyran-2-one interacted with the efflux pump from conventional hydrogen bonds with Thr201, Ser175, and Asn205; hydrophobic π-alkyl interaction with Ile55 and Ile40; and van der Waals interaction with several amino acids. As in our results with the NorA model, this coumarin, which presented the best result in silico, was not the most effective in vitro.

The types of interactions mentioned are relevant because they occur from structurally similar compounds and because conventional EPIs establish them. Verapamil, reserpine, piperine, CCCP, and CPZ interact with the NorA model, mainly through hydrophobic interactions (e.g., π-alkyl and π-π stacked), including with the same amino acids as C1 and C2, such as Phe16, Phe140, and Ile136. π-sulfur interaction (CPZ), carbon–hydrogen bonds (including with serine), and van der Waals interactions were also identified. Other amino acids, such as Ala48, Arg310, and Asn340, for example, are targets of EPIs and coumarins, but with different interactions [39,40].

The potential of coumarins as efflux inhibitors based on docking analyses with compounds known as EPIs is also related to the MepA efflux pump, considering that CCCP and CPZ establish conventional hydrogen bonds, carbon–hydrogen bonds, hydrophobic and van der interactions Waals with this model. Both EPIs establish π-sulfur interaction with the MepA model in the amino acid methionine, as identified with coumarin C1 [34,41].

According to the study by Chen, Li, and Li [42], the favorable interactions identified in molecular docking with coumarins make the binding of the molecules to the protein effective and increase the stability of the ligand-target complex. Despite the differences obtained between docking with the flexible and non-flexible receptors, the favorable interactions identified were similar, corroborating the potential of coumarins in inhibiting efflux pumps. Conversely, the steric bumps identified between C2 and the NorA model generate repulsion and instability in interactions, impairing the effect of the compounds [43]. This information corroborates our hypothesis about the slight difference between in vitro and in silico results.

In addition to the evidence that supports the hypothesis that the coumarins evaluated act as EPIs, we also demonstrated that C1 cannot alter the permeability of the cell membrane in strains 1199B and K2068, which reinforces the specificity of the mechanisms of these coumarins. This was confirmed by no change in the fluorescence intensity of SYTOX Green, which is an impermeable dye in living cells [44]. The opposite result was demonstrated by polymyxin B, an antibiotic known for its affinity to membrane components, such as phospholipids [45].

Zhong et al. [46] synthesized amphiphilic coumarins with hydrophobic and cationic chains linked to C-6 and C-7, respectively, and demonstrated that these compounds are active against standard and resistant strains of *S. aureus*, which occurs through effects on the cell membrane evidenced by the increase in SYTOX Green fluorescence. Similar results were obtained with coumarin aminophosphonates, using propidium iodide as the fluorescent compound [47]. The findings of these studies differ from our results due to the types of chemical groups added to the coumarin structure, which have properties related to the cell membrane [48,49].

## 4. Materials and Methods

### 4.1. Obtaining and Preparing Substances

The coumarins used were obtained through semi-synthesis from eugenol and supplied by the Pharmaceutical Chemistry Research Laboratory of the Federal University of Alfenas, located in Minas Gerais (Brazil). Details about the synthesis and characterization of the compounds are described in the study by Govêa et al. [50]. The coumarins evaluated were 3-benzoyl-8-methoxy-6-(prop-2-en-1-yl)-2*H*-chromen-2-one (C1), 8-methoxy-3-(4-nitrobenzoyl)-6-(prop-2-en-1-yl)-2*H*-chromen-2-one (C2), 3-(4-aminobenzoyl)-8-methoxy-6-(prop-2-en-1-yl)-2*H*-chromen-2-one (C3) and 8-methoxy-2-oxo-6-(prop-2-en-1-yl)-2*H*-chromene-3-carboxylic acid (C4) (Figure 1).

Antibiotics (norfloxacin and ciprofloxacin), ethidium bromide (EtBr), and efflux inhibitors: chlorpromazine (CPZ) and carbonyl cyanide 3-chlorophenylhydrazone (CCCP) were obtained from Sigma-Aldrich Co. (St. Louis, MO, USA). We diluted all compounds so that their solutions reached a concentration of 1024 μg/mL in the following ways: EtBr and CPZ diluted only in sterile distilled water, CCCP diluted in a solution (1:1) of sterile distilled water and methanol, and the others diluted in dimethyl sulfoxide (10%) and sterile distilled water [51,52].

### 4.2. Bacterial Strains and Inoculum Preparation

*Staphylococcus aureus* strains 1199B (overexpresses the NorA efflux pump) and K2068 (overexpresses MepA), provided by Prof. S. Gibbons (University of London, London, UK) and kept in stock at −20 °C in the Microbiology and Molecular Biology Laboratory of the Regional University of Cariri, were subcultured in Mueller Hinton Agar and incubated at 37 °C for 24 h before testing. The preparation of the inocula for use in antibacterial activity tests and association with antibiotics and EtBr occurred using McFarland’s 0.5 scale as a reference, following CLSI standards [53]. We prepared the inocula with phosphate-buffered saline (PBS) for the other assays.

### 4.3. Assessment of Antibacterial Activity

The evaluation of the antibacterial activity of coumarins and efflux inhibitors (CPZ and CCCP) occurred through broth microdilution (in Brain Heart Infusion Broth—BHI), determining the Minimum Inhibitory Concentration (MIC) of the compounds against *S. aureus* 1199B and K2068, according to Javadpour et al. [54]. The solutions prepared with 900 μL of BHI and 100 μL of inoculum were distributed in 96-well microplates (100 μL per well) and then microdiluted with the mentioned compounds (100 μL). Compound concentrations in the 96-well plates ranged from 512 to 8 μg/mL. We performed the assays in triplicate, and after 24 h of incubation (37 °C), the results were read using the colorimetric variation of sodium resazurin [55].

### 4.4. Association with Antibiotics and Ethidium Bromide

Coumarins, in subinhibitory concentration (MIC/8), were associated with antibiotics and EtBr against strains 1199B and K2068 of *S. aureus* following the methodology of Coutinho et al. [56]. EtBr (nonspecific substrate) was used against both strains to verify the activity of efflux pumps, while antibiotics (specific substrates) were used as follows: norfloxacin against *S. aureus* 1199B (NorA) and ciprofloxacin against *S. aureus* K2068 (MepA). The inhibitors CPZ and CCCP, also in subinhibitory concentrations, were used as a positive control. The tests took place in triplicate, and the reading followed the process described in the previous section.

### 4.5. Fluorescence Emission Test

According to the methodology of Da Silva et al. [57], we evaluated the effect of coumarin C1 on the fluorescence emission of EtBr by preparing the following solutions (in triplicate): (i) growth control: inoculum + PBS, (ii) negative control: inoculum + PBS + EtBr (100 μg/mL), (iii) positive control: inoculum + PBS + CCCP (50 μg/mL) + EtBr (100 μg/mL), (iv) C1 test: inoculum + PBS + C1 (10 μg/mL) + EtBr (100 μg/mL). After incubation for 1h30min, the solutions were centrifuged (10,000 rpm for 2 min), with the supernatant subsequently discarded. We then washed the material with PBS, centrifuging it again and discarding the supernatant. Finally, PBS was added to the pellets, and the solutions were distributed in microplates. We read the results from the BioTek^®^ Cytation 1 reader (Gen5™ 3.11 software), with wavelengths of 530 nm of excitation and 590 nm of emission.

### 4.6. Molecular Docking

Following the methods of Oliveira et al. [58] and Freitas et al. [59], the sequences of the NorA efflux pump from *S. aureus* 1199 (code Q03325) and MepA from *S. aureus* NCTC 8325 (code Q2G140), obtained from the UniProt database, were selected to build the three-dimensional models (3D) of proteins by homology, using the SWISS-MODEL server [60]. From the 50 templates available for each efflux pump, we chose the best model for each: PDB identifiers 7lo8 and 3wbn, respectively.

The ligand structures were prepared in the MarvinSketch^®^ software (Chemaxon©), version 23.11, and saved in 3D in the SDF format (Standard Database Format). Using the same software, we identified that two coumarins have ionized species: C3 (with NH_3_^+^ replacing the amino radical) and C4 (with O^−^ replacing the hydroxyl of the carboxyl radical), and these ionized forms were also evaluated.

The search for interactions between coumarins and efflux pump models by molecular docking took place in Molegro Virtual Docker, version 2019.7.0.0 [61]. The MolDock Score (score function) and the MolDock Optimizer (search algorithm) were used, keeping the other parameters as defaults, as described by De Lima Silva et al. [62]. We selected the central cavity of the efflux pump as the binding site. A radius of 15 Å was defined for the grid, using the center of the binding site as a reference (NorA coordinates: x: 140.37, y: 137.26, z: 150.16; MepA coordinates: x: 327.55, y: −10.16, z: 30.37). The best pose of each compound was selected based on the best values of interaction energy and MolDock Score.

In addition to carrying out molecular docking with flexible ligands, we also conducted evaluations with both flexible ligands and receptors, as carried out by De Araújo et al. [63]. To do this, we used the same coordinates mentioned in the previous paragraph and a grid box with dimensions of 30 Å × 30 Å × 30 Å. The analysis took place using AutoDock Vina tools (version 1.2.4), and we chose the poses corresponding to the best binding energies. The two-dimensional (2D) visualization of interactions took place in the BIOVIA Discovery Studio visualizer (Dassault Systèmes^®^), version 21.1.0. 20298, and in 3D in UCSF Chimera [64], version 1.17.3.

### 4.7. Effects on Membrane Permeability

The dye SYTOX Green (a DNA intercalator) was used for this test, as performed by Yuen et al. [65]. Initially, the inocula of *S. aureus* 1199B and K2068 were distributed in the 96-well black plate, and then, we added C1 coumarin until final concentrations of 200 µg/mL, 100 µg/mL, 50 µg/mL, and 25 µg/mL were obtained. Polymyxin B, at a concentration of 50 µg/mL, was used as a positive control, and the negative control contained only PBS. The plates were incubated for 1 h, and then, we added 100 µL of SYTOX Green at a final concentration of 1 µM. After another incubation for 1 h, a reading was performed with the same microplate reader and software mentioned above but with 485 nm excitation and 528 nm emission. We also performed the tests in triplicate.

### 4.8. Statistical Analysis

Statistical analysis was conducted using GraphPad Prism (version 8.0.1). The means of triplicates underwent analysis of variance (one-way ANOVA) and Bonferroni’s post-hoc test. Differences between the groups were significant when *p* < 0.05.

## 5. Conclusions

Given the potentiation of the activity of fluoroquinolones and EtBr, the increase in intracellular accumulation and fluorescence emission of EtBr, and the favorable interactions established in silico, it is concluded that the 3-substituted coumarins evaluated in the present research act as EPIs against NorA and MepA transporters of *S. aureus*. Among the compounds analyzed, coumarin C1 is the most promising, highlighting the impact of the benzoyl radical in the C-3 position on its effectiveness.

The absence of direct antibacterial effects against *S. aureus* 1199B and K2068, in addition to the lack of change in the permeability of the cell membrane, highlights the specificity of the mechanisms of these coumarins and their relevance as possible allies of antibiotic therapy in combating MDR strains through inhibition of efflux. Despite the positive results presented in this study, analyses are necessary to elucidate the potential and limitations of these coumarins. The results of this study imply more knowledge about the potential of coumarins, especially synthetic ones, against bacteria and antibiotic resistance, providing new information about structures/radicals/positions that can generate greater effectiveness in this type of biological activity so that this can be implemented in studies aimed at developing drugs capable of combating multiresistant bacterial strains carrying efflux pumps.

## Figures and Tables

**Table 1:** Minimum inhibitory concentration (MIC), in μg/mL, of compounds tested against *S. aureus* strains.

Compounds	SA 1199B	SA K2068
**C1**	≥1024	≥1024
**C2**	≥1024	≥1024
**C3**	≥1024	≥1024
**C4**	≥1024	≥1024
**CCCP**	64	64
**CPZ**	≥1024	≥1024

**Table 2:** Energies resulting from molecular docking between coumarins and the NorA efflux pump.

Coumarins	MolDock Score	Interaction	HBond	LE1
**C1**	−117.214	−129.417	−5.43272	−4.88392
**C2**	−125.394	−138.965	−10.5218	−4.64422
**C3**	−116.749 (−108.295)	−126.418 (−117.185)	−7.49231 (−0.38884)	−4.66997 (−4.33179)
**C4**	−99.4964 (−89.4395)	−109.64 (−99.1264)	−14.7348 (−5.6347)	−5.23665 (−4.70734)

HBond: hydrogen bond energy; LE1: efficiency of ligand 1 (MolDock score divided by the number of heavy atoms). Values in parentheses correspond to ionized species.

**Table 3:** Energies resulting from molecular docking between coumarins and the MepA efflux pump.

Coumarins	MolDock Score	Interaction	HBond	LE1
**C1**	−111.936	−121.81	−5	−4.66402
**C2**	−107.692	−119.787	−7.4925	−3.98861
**C3**	−109.268 (−108.887)	−118.995 (−119.946)	−2.5 (−4.59915)	−4.37073 (−4.35547)
**C4**	−83.2516 (−98.6581)	−92.5696 (−107.365)	−8.88168 (−7.5)	−4.38167 (−5.19253)

HBond: hydrogen bond energy; LE1: efficiency of ligand 1 (MolDock score divided by the number of heavy atoms). Values in parentheses correspond to ionized species.

## Data Availability

All data related to the study are included in the article.
